# Identification of COVID-19 and Dengue Host Factor Interaction Networks Based on Integrative Bioinformatics Analyses

**DOI:** 10.3389/fimmu.2021.707287

**Published:** 2021-07-28

**Authors:** Wenjiang Zheng, Hui Wu, Chengxin Liu, Qian Yan, Ting Wang, Peng Wu, Xiaohong Liu, Yong Jiang, Shaofeng Zhan

**Affiliations:** ^1^The First Clinical Medical School, Guangzhou University of Chinese Medicine, Guangzhou, China; ^2^The First Affiliated Hospital of Chinese Medicine, Guangzhou University of Chinese Medicine, Guangzhou, China; ^3^Shenzhen Hospital of Integrated Traditional Chinese and Western Medicine, Shenzhen, China

**Keywords:** COVID-19, dengue, co-infection, host factor interaction networks, bioinformatics analyses

## Abstract

**Background:**

The outbreak of Coronavirus disease 2019 (COVID-19) has become an international public health crisis, and the number of cases with dengue co-infection has raised concerns. Unfortunately, treatment options are currently limited or even unavailable. Thus, the aim of our study was to explore the underlying mechanisms and identify potential therapeutic targets for co-infection.

**Methods:**

To further understand the mechanisms underlying co-infection, we used a series of bioinformatics analyses to build host factor interaction networks and elucidate biological process and molecular function categories, pathway activity, tissue-specific enrichment, and potential therapeutic agents.

**Results:**

We explored the pathologic mechanisms of COVID-19 and dengue co-infection, including predisposing genes, significant pathways, biological functions, and possible drugs for intervention. In total, 460 shared host factors were collected; among them, CCL4 and AhR targets were important. To further analyze biological functions, we created a protein-protein interaction (PPI) network and performed Molecular Complex Detection (MCODE) analysis. In addition, common signaling pathways were acquired, and the toll-like receptor and NOD-like receptor signaling pathways exerted a significant effect on the interaction. Upregulated genes were identified based on the activity score of dysregulated genes, such as IL-1, Hippo, and TNF-α. We also conducted tissue-specific enrichment analysis and found ICAM-1 and CCL2 to be highly expressed in the lung. Finally, candidate drugs were screened, including resveratrol, genistein, and dexamethasone.

**Conclusions:**

This study probes host factor interaction networks for COVID-19 and dengue and provides potential drugs for clinical practice. Although the findings need to be verified, they contribute to the treatment of co-infection and the management of respiratory disease.

## Introduction

Coronavirus disease 2019 (COVID-19), which is due to novel severe acute respiratory syndrome coronavirus 2 (SARS-CoV-2), has led to a sudden and sharp increase in hospitalizations for pneumonia globally (1). In March 2020, the World Health Organization (WHO) publicly announced that COVID-19 had become a pandemic. As of June 22, 2021, there have been 178,503,429 confirmed cases of COVID-19 worldwide, including 3,872,457 deaths reported to the WHO, and the numbers continue to increase (https://covid19.who.int/).

Dengue, a mosquito-borne viral infection caused by four dengue virus serotypes (DENV 1-4), is found in tropical and subtropical climate areas, mainly in urban and semiurban regions (2). According to the WHO’s report, the global incidence of dengue has grown dramatically in the past few decades (https://www.who.int/news-room/fact-sheets/detail/dengue-and-severe-dengue). There are an estimated 100–400 million infections each year, and approximately half of the world’s population is now at risk of contracting the disease.

There is some cross-reactivity and common pathological processes, such as capillary leakage, thrombocytopenia, and coagulopathy, between SARS-CoV-2 and DENV (3, 4), making it difficult to distinguish their common clinical and laboratory characteristics. Overall, patients with dengue fever, including positive NS1 and/or IgM serology results, need to be differentiated from those with SARS-CoV-2 infection, and dengue IgM/IgG testing should be repeated if necessary to identify co-infection or serological overlap (5). Two patients in Singapore with false-positive results from rapid serological testing for dengue were later confirmed to have SARS-CoV-2 infection because of persistent fever, and the repeat dengue test (SD Bioline) was negative (6). This suggests that misdiagnosis between COVID‐19 and dengue is objective.

Serological overlap makes it difficult to identify symptoms, and co-infection complicates each disease. The COVID-19 pandemic and dengue fever recurrence epidemic in tropical countries have become global health issues. One case of co-infection has been reported in Mayotte, a French island in the Indian Ocean (7). Due to the delay in the diagnosis of COVID-19 and the transmission of dengue fever by the insect vector, measures need to be taken in endemic areas to avoid co-infection (8), and the diagnosis of patients with febrile disease should be organized to allow for diagnosis of both COVID-19 and dengue. In a research from Argentina, prolonged fever time was a clinical indication for suspected co-infection in most patients with mild COVID-19. Furthermore, in another study, headache was the most typical symptom in patients with co-infection (9). Colombia (10), Brazil (11), Pakistan (12), and other countries have reported that COVID-19 and dengue may cause overlapping epidemics and increase the number of critically ill patients, thereby compounding the burden on the healthcare system. There are overlapping outbreaks of COVID-19 and dengue in dengue-endemic countries. Therefore, identifying potential vector breeding sites to protect people from mosquito bites and maintaining social distancing to avoid the risk of COVID-19 transmission are very important (13).

It is necessary to prepare for dengue outbreaks immediately under the premise of controlling the COVID-19 pandemic. The commissioners of Lancet Commission on Dengue and Arboviral Diseases announced that an approach should be adopted for the prevention and detection of dengue and other arboviral diseases during the COVID-19 pandemic in tropical and subtropical regions (14). Co-infection of SARS-CoV-2 and dengue has become a hot research topic, and the common pathogenesis and biological pathways of the two can be used as resection points for project research.

In our study, we compared SARS-CoV-2 and DENV by bioinformatics analysis and obtained 460 common core targets and 30 common critical pathways through Gene Ontology and KEGG enrichment to identify host factor interaction networks between the two viruses. More in-depth and more comprehensive analyses of enormous amounts of data on RNA viruses such as SARS-CoV-2 and DENV can reveal the molecular mechanisms by which viruses infect hosts and can also help in designing therapies.

## Materials and Methods

### Collection of COVID-19-Associated Genes

COVID-19-related targets were gathered from GSE147507, GSE155249, and GSE157103 by searching the Gene Expression Omnibus (GEO) database (https://www.ncbi.nlm.nih.gov/geo). GEO is an international public repository that archives and freely distributes microarrays, next-generation sequencing, and other forms of high-throughput functional genomics data submitted by the research community. The GSE147507 dataset involves the transcriptional response to SARS-CoV-2 infection by expression profiling with high-throughput sequencing and detailed transcriptomic analysis of blood, lung, and bronchus samples from COVID-19 cases (15). The GSE155249 dataset recorded circuits between infected macrophages and T-cells in SARS-CoV-2 pneumonia through high-throughput sequencing of alveolar lavage fluid (16). The GSE157103 dataset entails a large-scale and multiomics analysis of the severity of COVID-19 (17). We obtained information by analyzing a set of differentially expressed genes (DEGs) from the above three datasets and screened the host gene set at FDR (false discovery rate) <0.000001 and | LogFC |≥1.

In addition, we screened COVID-19-related host factors through PubChem (https://pubchem.ncbi.nlm.nih.gov/#query=covid-19), Comparative Toxicogenomics Database (CTD) (http://ctdbase.org/), and DisGeNET (https://www.disgenet.org/covid/diseases/summary/). PubChem is a public repository for archiving biological data of small molecules obtained through high-throughput RNA interference screening, aimed at identifying critical genes responsible for a biological process or disease condition (18). CTD is a public resource for literature-based methods to store disparate information for toxicogenomics, gene expression profiles, diseases, environmental exposures, and pharmaceuticals (19). DisGeNET is a wide-ranging platform that integrates data on the genetic basis of human diseases and collects data on genotype-phenotype relationships from several resources, including Mendelian, complex, environmental and rare diseases, and disease-related traits (20). The top 500 hits in each database were included. The date of access to the above six databases was December 10, 2020.

### Collection of Dengue-Associated Genes

We obtained dengue-related genes from the results of expression profiling by high-throughput sequencing, including GSE38246, GSE51808, and GSE84331, from GEO. The criteria for screening host genes for each data set included FDR <0.000001 and | LogFC | ≥ 1. We searched CTD (http://ctdbase.org/), DisGeNET (https://www.disgenet.org/), and GeneCards (https://www.genecards.org/) at the same time and screened the top 500 hits in the three databases. Among them, the GSE38246 (21), GSE51808 (22), and GSE84331 (23) datasets provide host factors required for DENV replication. GeneCards is an authoritative compilation of annotative information that provides concise genome, proteome, transcriptome, disease, and function data on all known and predicted human genes (24). The date of access to the above six databases was December 10, 2020.

### Protein-Protein Interaction Analysis and Network Construction

Common host factors between COVID-19- and dengue-related targets were identified using the web tools provided by the Van de Peer Lab (http://bioinformatics.psb.ugent.be/webtools/Venn/) and input into the STRING website (version 11.0, https://string-db.org/) (25) to generate a protein-protein interaction (PPI) network; the top 50 genes were selected. The PPI results were analyzed and visualized using Cytoscape-3.8.1 (https://cytoscape.org/) (26, 27). CytoHubba (http://apps.cytoscape.org/apps/cytohubba) (28) was applied to perform network topology analysis. Then, module analysis of the PPI network was performed by Molecular Complex Detection (MCODE) in Metascape (https://metascape.org/) (29) and exported to Cytoscape-3.8.1 for further visualization processing, making the module concise and clear.

### Gene Ontology and Pathway Enrichment Analysis

We used R software to perform Gene Ontology and KEGG pathway enrichment analyses of genes in common host factor interaction networks. We chose a two-sided hypergeometric test and Bonferroni step down for P-value correction (P < 0.05). The ShinyGO v0.61 platform (http://bioinformatics.sdstate.edu/go/) was used for WIKI pathway enrichment analysis.

### Inference of Upstream Pathway Activity

SPEED2 (https://speed2.sys-bio.net/) (30) is a signaling pathway enrichment analysis tool that is used to presume upstream pathway activity of the host factor interaction networks between SARS-CoV-2 and DENV. We first applied a list of gene IDs and then chose the Bates test for test enrichment statistics. When clarifying transcriptome data, it is important to infer which signaling pathway triggers a particular gene expression program rather than to score signaling pathway activity.

### Tissue-Specific Enrichment Analysis of Top Genes

Genotype-Tissue Expression (GTEx) was performed by using the GTEx Project (https://www.gtexportal.org/home/), which will help researchers understand inherited susceptibility to disease by identifying regions of the genome that influence whether and how much a gene is expressed (31, 32). Tissue-specific enrichment calculates tissue-specific gene enrichment in an input gene set to assign tissue identities to single-cell clusters and differentiated embryonic stem cells. We used R software to perform tissue-specific enrichment analysis.

### Predicting Drugs Through Interaction Networks of Chemicals and Proteins

STITCH (search tool for interactions of chemicals) integrates information about drug-target relationship information for over 68,000 different chemicals, including 2,200 drugs, and connects them to 1.5 million genes (http://stitch.embl.de/). We used STITCH to predict related drugs that might provide intervention or treatment for these diseases.

## Results

### Identification of Common Host Factors Between COVID-19 and Dengue

After searching for COVID-19 based on CTD, we retrieved 7,726 related host genes. By searching COVID-19 in DisGeNET, 1,843 related genes were identified. Similarly, we obtained 651 genes by searching COVID-19 in PubChem. The above data were selected from the top 500 genes in accordance with relevance scores. From three transcriptomics analyses, 154 factors were obtained from GSE147507. In addition, 168 host factors were obtained from GSE155249, which was the result of the study about bronchoalveolar lavage fluid samples. In total, 202 constituents were acquired from GSE157103, which was the result of the study about plasma and leukocyte samples. The selection criteria were FDR<0.000001 and |LogFC|≥1. Positive logFC indicates the logarithmic degree of upregulation, and negative logFC indicates the logarithmic degree of downregulation. Eventually, we included 1,721 host-related genes by collecting the union of the genes collected in the database and the host factors analyzed by transcriptomics.

On the basis of searches in CTD, DisGeNET, and GeneCards, we identified 2,679, 496, and 110 targets relevant to DENV, respectively. Among them, the top 500 from CTD were included, and the data form the other two were all included. Based on transcriptome data analysis of GSE38246, GSE51808, and GSE84331, 1, 845, and 276 related host factors, respectively, were identified.

**Figure 1:**
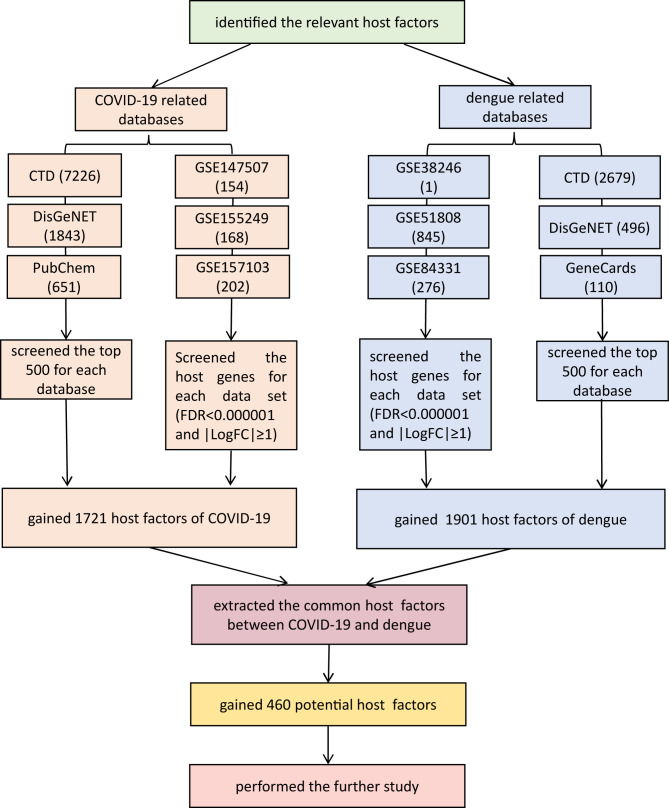
The screening process of shared targets between COVID-19 and dengue. In total, 460 factors were shared. False discovery rate (FDR) using a Benjamini-Hochberg approach (FDR<1×10^-6^) and |logFC| ≥ 1. Venn diagram tool (http://bioinformatics.psb.ugent.be/webtools/Venn/). CTD: Comparative Toxicogenomics Database (http://ctdbase.org/). DisGeNET: a platform containing genes associated with human diseases (https://www.disgenet.org/). PubChem: a collection of accessible chemical information (https://pubchem.ncbi.nlm.nih.gov/). GeneCards: a database of human genes (https://www.genecards.org/). GEO: Gene Expression Omnibus (https://www.ncbi.nlm.nih.gov/geo/).

We identified 1,721 COVID-19-associated targets and obtained the gene names of 1,901 dengue-related genes, with no repetition. After collating COVID-19- and dengue-associated targets, these two target categories were compared and filtered to identify common elements using the web tools provided by the Van de Peer Lab. Ultimately, 460 common host factors were obtained, including MMP2, PDF, PFKP, SLC25A3, IGF1, CCL4, TLR4, and AhR. The screening process of shared targets between COVID-19 and dengue is shown in **Figure 1**.

### PPI Network and Module Analysis

**Figure 2:**
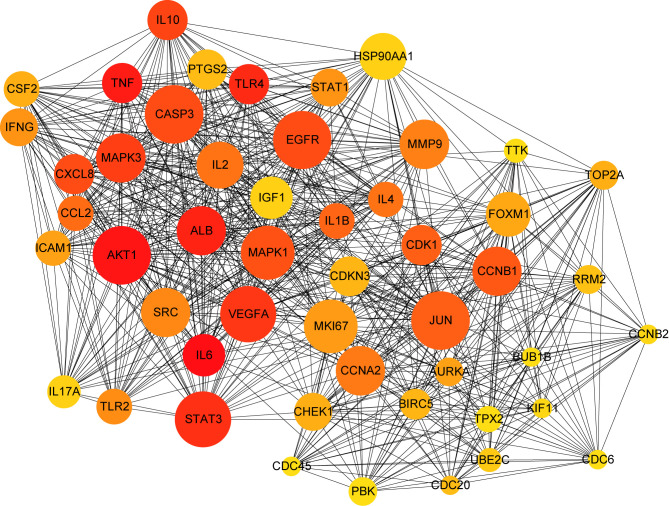
PPI network of common targets between COVID-19 and dengue. In the figure, the circle nodes indicate genes, and edges specify interactions between nodes.

**Figure 3:**
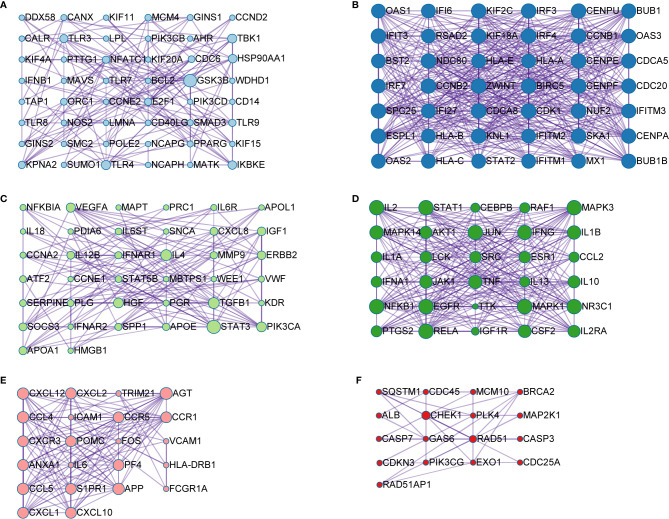
Module analysis of the 460 shared genes. Each module demonstrates different biological process functions. **(A)** module 1; **(B)** module 2; **(C)** module 3; **(D)** module 4; **(E)** module 5; **(F)** module 6.

A PPI network was produced by STRING based on common host factors and reimported into Cytoscape-3.8.1 for visual analysis and network topology. The order of degree value from high to low was IL-6, AKT1, TNF, ALB, TLR4, and STAT3, among others as shown as **Figure 2**. Through MCODE of Metascape, we identified highly interconnected portions and screened 11 vital modules on the basis of interaction among the 460 targets. The degree of the node can be seen intuitively in **Figure 3**. The greater is the number of points, the higher is the degree. In module 1, the hub nodes were GSK3B, TLR4, TBK1, IKBKE, and HSP90AA1. In module 2, the hub nodes were BUB1, BUB1B, CENPA, and BIRC5. In module 3, the hub nodes were STAT3, IL-4, TGFB1, HGF, and PIK3CA. In module 4, the hub nodes were NFKB1, EGFR, and JUN. In module 5, the hub nodes were AGT, APP, CCL4, CCL5, and CXCL12. In module 6, RAD51 was the most important target.

### Gene Ontology and KEGG Pathway Enrichment Analyses

**Figure 4:**
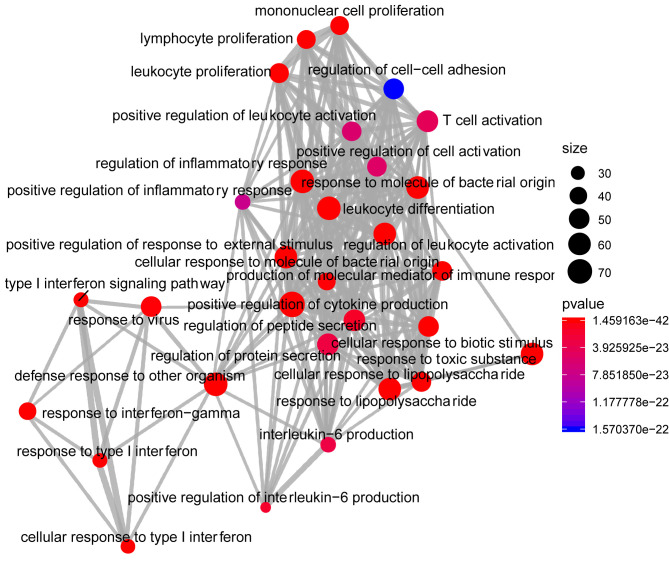
Network of enrichment analysis of GO biological process terms. The size of the node represents the number of genes involved in the enrichment pathway, and the color from blue to red indicates the P-value from small to large.

**Figure 5:**
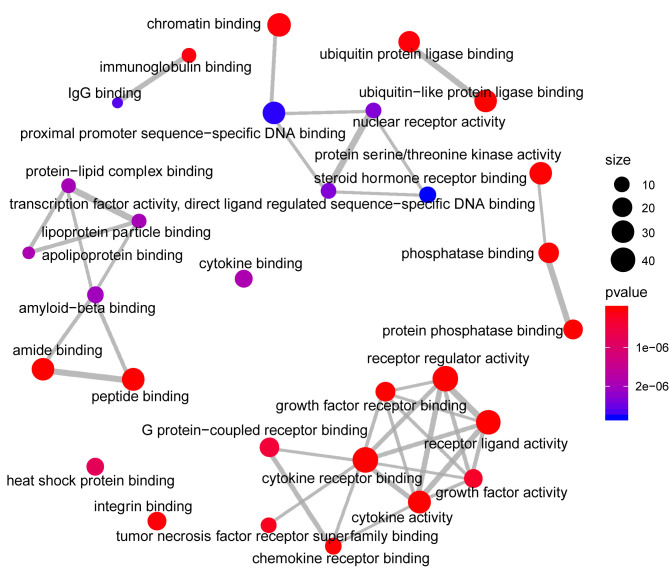
Network of enrichment analysis of GO molecular function terms. The size of the node represents the number of genes involved in the enrichment pathway, and the color from blue to red indicates the P-value from small to large.

**Figure 6:**
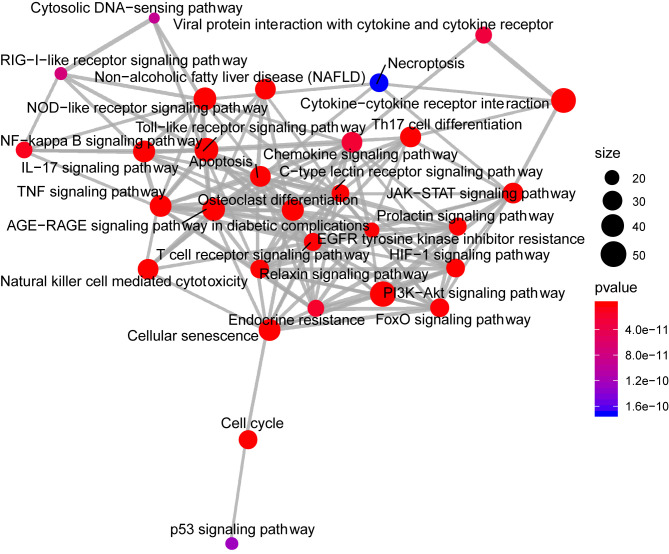
The network of pathway enrichment analysis through KEGG. The size of the node represents the number of genes involved in the enrichment pathway, and the color from blue to red indicates the P-value from small to large.

Analysis of the Gene Ontology biology process in the **Figure 4** showed that genes of the host networks are related to the process of occurrence and development, including Response to stress, Response to organic substance, Response to cytokine, Cellular response to organic substance, and Cellular response to chemical stimulus, as well as other cell regulation of response to external pathogen stimulation. In addition, molecular function analysis in **Figure 5** revealed signaling receptor binding, identical protein binding, enzyme binding, cytokine receptor binding, drug binding, and others. According to KEGG pathway enrichment analysis, influenza A, measles, hepatitis B, hepatitis C, pathways in cancer, Epstein-Barr virus infection, and the Toll-like receptor (TLR) signaling pathway play important roles in interaction between COVID-19 and dengue, that shows in **Figure 6**. The TLR Signaling Pathway, Regulation of toll-like receptor signaling pathway, Spinal Cord Injury, AGE/RAGE pathway, Oncostatin M Signaling Pathway, PI3K-Akt Signaling Pathway, and Photodynamic therapy-induced NF-kB survival signaling in WIKI were important biological function pathways in the host factor interaction networks, as shown in **Table 1**.

**Table 1:** Results of pathway enrichment analysis through WIKI.

Enrichment FDR	Genes in list	Total genes	Functional Category
5.57E-42	42	102	Wiki : Toll-like Receptor Signaling Pathway
2.10E-38	44	139	Wiki : Regulation of Toll-like receptor signaling pathway
1.22E-29	35	116	Wiki : Spinal Cord Injury
1.51E-28	28	66	Wiki : AGE/RAGE pathway
2.72E-27	27	65	Wiki : Oncostatin M Signaling Pathway
4.91E-26	49	339	Wiki : PI3K-Akt Signaling Pathway
1.03E-25	21	35	Wiki : Photodynamic therapy-induced NF-kB survival signaling
4.16E-25	41	234	Wiki : VEGFA-VEGFR2 Signaling Pathway
6.67E-25	28	87	Wiki : Retinoblastoma (RB) in Cancer
9.60E-25	23	50	Wiki : Photodynamic therapy-induced AP-1 survival signaling
5.57E-24	26	76	Wiki : Leptin signaling pathway
1.72E-23	27	88	Wiki : Allograft Rejection
1.05E-21	21	50	Wiki : Vitamin B12 Metabolism
1.05E-21	21	50	Wiki : Hepatitis C and Hepatocellular Carcinoma
1.05E-21	19	37	Wiki : Fibrin Complement Receptor 3 Signaling Pathway
1.44E-21	23	66	Wiki : Folate Metabolism
1.61E-21	25	84	Wiki : Apoptosis
2.12E-21	24	76	Wiki : Prolactin Signaling Pathway
2.12E-21	27	105	Wiki : Senescence and Autophagy in Cancer
4.83E-21	16	24	Wiki : IL1 and megakaryocytes in obesity
8.82E-21	25	90	Wiki:T-Cell antigen Receptor (TCR) Signaling Pathway
1.54E-20	25	92	Wiki : Corticotropin-releasing hormone signaling pathway
1.67E-20	28	125	Wiki : Ebola Virus Pathway on Host
2.80E-20	16	26	Wiki : Cytokines and Inflammatory Response
3.23E-20	24	85	Wiki : Selenium Micronutrient Network
1.02E-19	19	46	Wiki : Aryl Hydrocarbon Receptor
1.02E-19	24	89	Wiki : Pancreatic adenocarcinoma pathway
1.28E-19	20	54	Wiki : IL-4 Signaling Pathway
4.30E-19	18	42	Wiki : TNF related weak inducer of apoptosis (TWEAK) Signaling Pathway
4.30E-19	18	42	Wiki : IL-2 Signaling Pathway

### Upstream Pathway Activity

**Figure 7:**
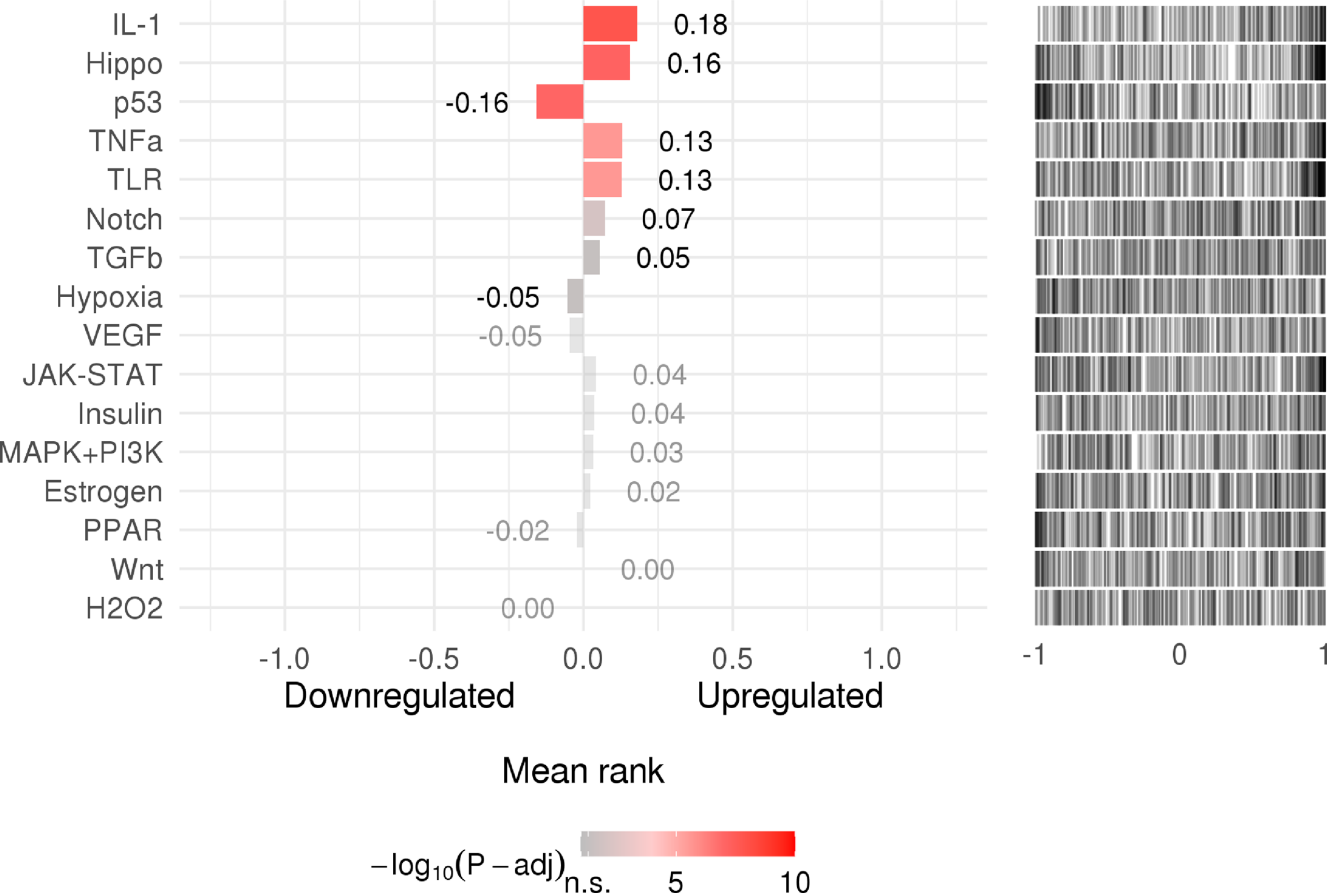
The rank of signaling pathway activity (adjusted P-value <0.05). The color represents the adjusted P-value; the brighter the color, the higher the rank.

SPEED2 is a signaling pathway annotation enrichment analysis tool with gene sets derived from pathway perturbation biological experiments in human cell lines. The understanding of incorporated regulatory mechanisms can be simplified by identifying modulated pathways upstream of differentially expressed genes. Z-values, which we refer to as Zrank, were rescaled for each experiment to the interval, providing the average signed-rank for this gene across those experiments. If the Z-value was higher than zero, the corresponding gene was upregulated; if it was lower than zero, it was downregulated. Then, its significance was assessed by a P-value, and ranked lists covering all of the measured genes were used to assess enrichment or depletion of a user-provided gene list. IL-1, Hippo, p53, TNF-α, and TLR occupied a pivotal position as shown in as shown in **Figure 7**.

### Tissue-Specific Enrichment Analysis of Host Factor Interaction Networks

**Figure 8:**
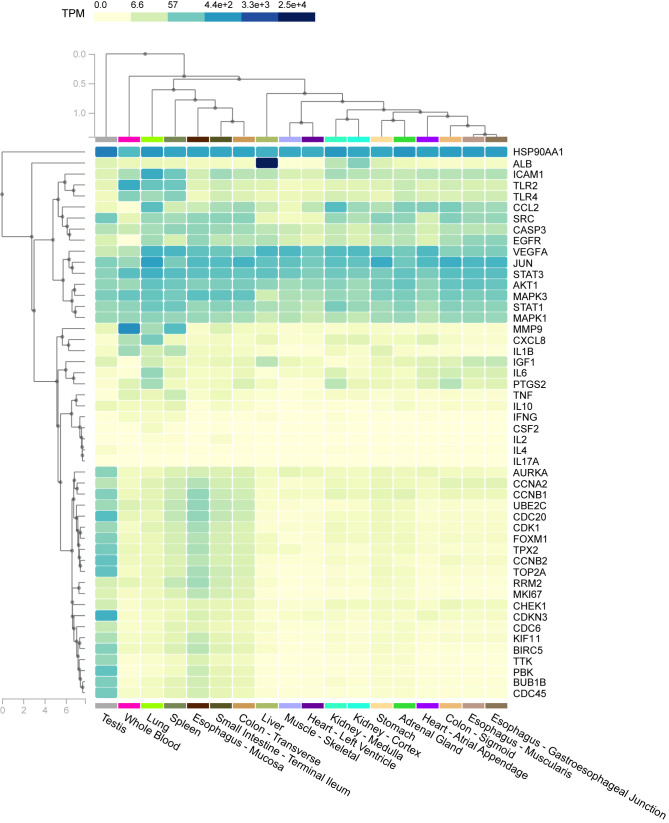
The heat map shows the correlation between different samples and factors. Columns represent genes, rows represent tissues, and colors represent the level of enrichment, whereby a darker color indicates a more significant gene in corresponding tissue.

In tissue-specific enrichment analysis, each column represents a different tissue, and each line represents a different factor; the higher is the value of TPM, the darker is the color, and the density of specific distribution of host factors in corresponding tissue is higher. The heat map in **Figure 8** shows the correlation between different samples and factors. For example, the specific distributions of host factors in the lung included HSP90AA1, ICAM1, and JUN.

### Predicted Drugs Through Host Target Interactions With Chemicals

STITCH aims to construct a database that integrates data in the literature and various databases of biological pathways, drug-target relationships, and binding affinities. We used it to predict pharmaceutical preparations according to chemical protein interactions and drug-target relationships. The drugs related to most genes mainly involve retinoic acid, estradiol, dexamethasone, and estrogen. The list of suggested agents for COVID-19 and dengue co-infection can be seen intuitively in **Table 2**. Among them, retinoic acid had the most enriched targets, with 471 genes and 84 genes on the list. Resveratrol, an anti-inflammatory and antioxidant, showed 180 genes in its enrichment target, of which 64 were on the list.

**Table 2:** List of suggested agents for COVID-19 and dengue co-infection.

Enrichment FDR	Genes in list	Total genes	Functional Category
3.90E-59	64	180	resveratrol(CID000445154)
4.01E-58	68	223	PD98059(CID000004713)
8.09E-58	59	150	SB203580(CID000176155)
2.02E-57	67	221	genistein(CID005280961)
2.02E-57	59	153	p-38(CID100200878)
4.59E-57	60	163	resveratrol(CID100005056)
1.75E-56	83	410	dexamethasone(CID000005743)
1.75E-56	55	129	SB203580(CID100176155)
4.23E-55	63	201	LY294002(CID000003973)
6.22E-54	57	157	p-38(CID000200878)
6.22E-54	62	200	PD98059(CID100004713)
1.14E-52	84	471	retinoic acid(CID000444795)
2.00E-52	80	419	retinoic acid(CID100005538)
1.99E-51	73	340	dexamethasone(CID100003003)
1.67E-50	53	144	curcumin(CID000969516)
1.79E-50	58	187	genistein(CID105280961)
4.16E-50	80	449	estradiol(CID000005757)
4.56E-49	75	393	estrogen(CID000448537)
4.76E-49	73	367	estradiol(CID100000450)
2.68E-48	61	233	cycloheximide(CID000006197)
3.12E-48	55	175	LY294002(CID100003973)
6.38E-48	49	127	curcumin(CID100002889)
1.70E-47	65	285	phorbol ester(CID010282255)
9.32E-45	58	231	nitric oxide(CID000000945)
3.35E-44	54	194	nitric oxide(CID100000945)
1.10E-43	65	325	estrogen(CID100003054)
1.95E-43	65	328	ethanol(CID000000702)
1.28E-41	50	174	quercetin(CID005280343)
3.66E-40	42	113	U0126(CID100005637)
4.59E-40	54	229	phorbol ester(CID110282255)

## Discussion

Both COVID-19 and dengue are international public health issues with adverse effects, and co-infection exacerbates the worldwide burden on healthcare systems. Moreover, the similar clinical manifestations and serological overlap between COVID-19 and dengue raise the risk of incorrect diagnosis. There is also a possibility that overlapping immunological cascades may affect not only disease severity but also vaccine development (3). In view of this condition, we analyzed host factor interaction networks of COVID-19 and dengue co-infection from the perspective of biological pathways and attempted to screen potential agents. To elucidate mechanisms, we identified common host factors and performed further analysis. A PPI network was created, and vital modules were analyzed. Subsequently, biological functional analysis with Gene Ontology terms, KEGG, and WIKI was completed. In addition, signaling pathway activity was assessed, and tissue-specific enrichment analysis was carried out. Finally, candidate drugs were detected, providing a basis for in-depth research on therapeutic strategies.

This study involved multiple steps. On the one hand, the approach illustrated the role of pathogens in disease development processes with a progressive logical relationship, echoing the biological effects of viruses. The collection source combined the database with transcriptome data, and the method combined union and intersection, making the dataset more comprehensive and accurate. On the other hand, the multiple steps helped in overcoming the limitations of cross-sectional, single-scale, and one-dimensional studies. Dynamic evolution in the host during processes of infection and immune response were analyzed at multiple scales, such as factors, pathways, and tissue, reflecting a dynamic, multiscale, and multidimensional research perspective. This study involved seven separate sections each with its own distinct method and outcome, yet the whole was integrated and complete.

### Identification of Common Host Factors Between COVID-19 and Dengue

After comparing the collected genes, 460 host factors common to COVID-19 and dengue were found, including MMP2, PDF, PFKP, SLC25A3, IGF1, CCL4, TLR4, and AhR, and further analysis of interaction networks was performed.

COVID-19 pathogenesis is associated with excessive cytokine release, such as CCL4 (33). This factor can dominate the chemokine signature and shows persistently high concentrations in patients with severe COVID-19 (34). Moreover, massive cytokine secretion is regarded as a part of the underlying mechanism in dengue. As shown by a study from Mathieu Nacher, dendritic cells infected by DENV express CCL4, which is associated with vasodilation, endothelial dysfunction, and disease severity (35). In SARS-CoV-2-infected cells, overproduced plasminogen activator inhibitor-1 (PAI-1) binds to TLR4 on macrophages, inducing secretion of proinflammatory cytokines and chemokines (36). For patients with dengue, TLR4 on platelets binding with DENV NS1 is triggered, causing thrombocytopenia and hemorrhage (37).

The shared factors are thought to be involved in other activities as well. In COVID-19 patients, AhR stimulation upregulates expression of mucins in alveolar epithelial cells, causing accumulation of alveolar mucus and leading to silent hypoxia formation, a unique feature of the disease (38). The role of factors in DENV replication was also investigated. It has been reported that an increase in DENV titer is related to activated AhR; conversely, replication is inhibited by an AhR antagonist. In summary, AhR appears to promote DENV replication (39).

### PPI Network and MCODE Analyses Revealed Key Factors of Host Factor Interaction Networks

Targets with high interaction rates in host factor interaction networks were obtained from PPI network analysis and might be treatment targets, including IL-6, AKT1, TNF, ALB, TLR4, STAT3, and JUN, among others.

IL-6 is elevated in COVID-19 and dengue and is related to the severity of both (40). It can induce the production of other inflammatory cytokines by interacting with many different cells and can cause E-cadherin expression; this leads to increased endothelial permeability, which may result in Acute Respiratory Distress Syndrome (ARDS), a fatal comorbidity of COVID-19 (41, 42). As reported by M Juffrie (43), this process is associated with plasma leakage and shock caused by increased permeability in dengue patients. Previous research has proposed that IL-6 contributes more to the disease pathogenesis of COVID-19 than does DHF, and serum IL-6 levels are higher in severe pneumonia patients with COVID-19 (44).

Virus-induced apoptosis is a significant event that determines pathogenicity and virulence, though studies on the function of JUN in SARS-CoV-2-induced apoptosis are limited. According to a study on IBV (avian coronavirus infectious bronchitis virus), c-JUN seems to protect coronavirus-infected cells from apoptosis (45). Interestingly, in contrast to c-JUN, the findings from several studies suggest that the c-Jun N-terminal kinase (JNK) pathway exerts a crucial effect on regulating apoptosis evoked by coronavirus infection (45–47). In a study of colony transcriptomes (48), c-JUN was highly expressed in DENV2-refractory colonies. In addition, the findings from a study about JNK by Avisha Chowdhury suggest that the pathways have a broad antiviral function against DENV, depending on apoptosis induction and the complement system. Moreover, activated c-JUN participates in controlling the rearrangement of membrane structures, which is induced by DENV and required for replication (49).

In total, 11 different plates were obtained with the MCODE module in analysis of 460 common host factors. The main function reflected by plate B was interferon (IFN) signaling.

Both SARS-CoV-2 and DENV can promote activation of immune cells, leading to the release of proinflammatory cytokines, such as IFN, and causing capillary leakage, thrombocytopenia, and coagulopathy, pathophysiological similarities between COVID-19 and dengue (3). In a study of SARS-CoV-treated mice, interleukins, IFN, and chemokines were notably increased within 24 h (50). Among them, IFNs have antiviral, antiproliferative, and immunomodulatory functions and are regarded as the first line of the body’s antiviral defenses (51). Nevertheless, pDCs and monocytes produce more IFN than other cells after viral infection, and innate immune response plays a significant role in the formation of the cytokine storm (50). In addition, IFN‐γ in combination with other inflammatory mediators can facilitate plasma leakage, disseminated intravascular coagulation (DIC), and other vascular disorders (3), which is the main pathological characteristic of dengue.

### Enrichment Analysis Showed the Biological Functions of Host Factor Interaction Networks

To investigate interaction networks at the molecular level, functional enrichment analysis, including functional annotations of significant factors and pathway enrichment analysis, was carried out. GO was utilized to classify common factors. In this study, the classification was executed according to biological process and molecular function categories. Some targets were identified as being associated with the response to stress, response to an organic substance, response to cytokine in biological processes, and response to signaling receptor binding, identical protein binding, enzyme binding, and cytokine receptor binding in molecular functions. The cytokine response was significant.

SARS-CoV-2 infection gradually induces an excessive inflammatory response and dysregulated immune defense, which results in tissue damage and causes acute lung injury (ALI) and even ARDS. The mechanism may be due to the massive release of cytokines and chemokines, which might be a characteristic of COVID-19 (33). The cytokine storm triggers plasma leakage and disseminated vascular coagulation, which are life-threatening respiratory symptoms in COVID-19 (52).

The pathogenesis of dengue includes cytokine storms, vascular leakage, complement activation, autoimmunity, and antibody-dependent enhancement. Through research on patients with dengue hemorrhagic fever or dengue shock syndrome, it was observed that cytokines are produced and change rapidly during the course of the illness. T cells, macrophages, monocytes, and mast cells can produce several soluble factors, such as TNFα, IL-6, IL-8, and IL-10, to increase the vascular permeability of primary endothelial cells (2).

Through KEGG and WIKI, we analyzed the biological function of pathways in host factor interaction networks, such as influenza A, measles, and hepatitis B in KEGG and the TLR signaling pathway, regulation of Toll-like receptor signaling pathway, and spinal cord injury in WIKI.

According to the findings of this study, the TLR pathway has an important position, as revealed by KEGG and WIKI. After the viral spike protein binds to host cells *via* ACE2, SARS-CoV-2 RNA is detected by TLRs (52). Similarly, TLRs recognize DENV; TLR3 is able to establish an antiviral state (53), and TLR 4 can be utilized by DENV to activate platelets *in vitro* (54).

Based on WIKI, another pathway shared by COVID-19 and dengue is the NOD-like receptor signaling pathway. NLRP3 is a member of the pathway, and its activation by Viroporin 3a seems to have an important effect on the pathogenesis of SARS-COV infection (55). According to relevant studies, SARS-CoV-2 infection causes a range of disease manifestations, with the most serious being a massive inflammatory response that appears to occur *via* activation of the NLRP3 inflammasome (56). Dengue is related to the processing and release of proinflammatory cytokines. After infection, the NLRP3-specific inflammasome is stimulated by DENV through inflammasome activation. According to the present study, the assembly process of the NLRP3 inflammasome complex is promoted by the DENV-2 NS2A and NS2B proteins. NLRP3 further exerts its effect on IL-1β by regulating its maturation and secretion (57), increasing endothelial permeability and causing vascular leakage (58).

### Pathway Activity and Tissue Specificity of Host Factor Interaction Networks

In this study, we also assessed the signaling activity of dysregulated host factors, which is important to infer disease mechanisms. According to the results, upregulated genes include IL-1, Hippo, and TNF α, among others.

IL-1B, a proinflammatory cytokine, plays a vital role in the inflammatory response caused by infectious pathogens (59). IL-1B is activated in severe COVID-19, inducing vasodilation and permeability and providing the conditions for immune cells to arrive at the sites of damage. In addition, it can induce complement activation and opsonization (60). The production of IL-1B is promoted by platelets after infection with DENV, and the synthesis of IL-1B-containing microvesicles is induced by DENV, which ultimately increases vascular permeability (61). Moreover, IL-1B-31C carriers run the risk of dengue shock syndrome (DSS), implying that IL-1B is associated with the pathogenesis of DSS (62).

TNF-α is a vital driver of inflammation (63). According to relevant mouse studies *in vitro* and *in vivo*, synergistic activity of IFN-γ and TNF-α mimics the symptoms of COVID-19 and triggers robust cell death (64). Moreover, TNF receptor levels have been reported to show a positive correlation with dengue hemorrhagic fever (DHF) severity (65). A study of a mouse model of dengue hemorrhage implied that TNF-α induces hemorrhage, resulting in endothelial cell death (66, 67).

To explore the novel association of tissue with genes in interaction networks, tissue-specific enrichment analysis was performed. For example, expression of ICAM-1 was highly enriched in the lung.

Overall, the investigation of endothelial cell adhesion molecules is meaningful for understanding the pathophysiology of COVID-19. As reported by previous research, expression of adhesion molecules, such as ICAM-1 and VCAM-1, is related to systemic inflammation and might contribute to coagulation dysfunction (68). In dengue, increased expression of ICAM-1 can result in the consequent influx of cells promoting inflammation of the endothelium. Relevant studies are in favor of the development of new markers for the evaluation, therapeutic response, and clinical follow-up of patients with severe dengue (69). Thus, ICAM-1 might be important for co-infection.

### Identification of Candidate Drugs Against Co-Infection

Finally, we studied chemical-protein interaction networks with STITCH to identify several candidate agents with the potential to treat COVID-19 and dengue co-infection, such as resveratrol, curcumin, and quercetin. The findings require appropriate validation. For example, quercetin has good antiviral, immune modulation, and anti-inflammatory properties and is a potential agent for treating co-infection. However, quercetin is genetically toxic, mutagenic, and has other insuperable side effects (70), but it provides some guidance for drug development for the treatment of co-infection.

Resveratrol is a well-known anti-inflammatory and antioxidant agent (71). It can act on several mechanisms of COVID-19, as shown by the research of Anderson O. Ferreira. The drug can reduce entry of the virus by blocking binding to ACE2 (72). Furthermore, it reduces NF-kB, inhibits the TLR4 pathway, and decreases the production and expression of some significant inflammatory factors (73). As an endothelial barrier protector, it can protect cells from stressful conditions to weaken endothelial inflammation (74). Moreover, it has a potential antithrombotic effect (75). With regard to dengue, the drug can inhibit HMGB1 translocation to increase activation of ISGs and subsequently exert an effect by partially suppressing DENV replication (71).

## Limitations

However, there are some limitations in this study. First, the measurement methods for genomic data differed among studies, which made it difficult to achieve complete unity. Second, in view of the limitation of disease-gene and drug-target bipartite networks in network pharmacology, associated information loss was inevitable (76). Furthermore, the analysis methods were dependent on computational power and data acquisition (77), which was based on current research; hence, it was difficult to predict the complete mechanism. Third, the potential of resveratrol, a well-known anti-inflammatory and antioxidant agent, to treat co-infection with COVID-19 and dengue was based on data mining–based bioinformatics tools, and experimental validation is required.

## Conclusion

In this study, mechanisms of co-infection were preliminarily revealed in terms of host factor interaction networks. With a series of methods, the core factors and pathways were screened, the biological function was analyzed, and new ideas for treatment were revealed. However, as this study was based on data mining and analysis, there were limitations; thus, more validated and rigorous experiments are required to verify our prediction.

## Data Availability Statement

The data used to support the findings of this study are available in 4TU.RearchData (DOI: https://doi.org/10.4121/14769018).

## Author Contributions

WZ conceived and designed the study and plotted the figures based on network bioinformatics, using online databases. HW and CL conducted data analysis and performed literature searches. QY, TW, and PW carried out literature searches and studies of the background of the disease. XL, YJ, and SZ reviewed and revised the manuscript. All authors contributed to the article and approved the submitted version.

## Funding

This research was funded by grants from the “Double First-Class” and High-level University Discipline Collaborative Innovation Team Project of Guangzhou University of Chinese Medicine (Grant No.2021XK16), Guangdong Provincial Department of Education Innovation Team Project (Grant No: 2018KCXTD007), the Key-Area Research and Development Program of Guangdong Province (Grant No. 2020B1111100002), the National Natural Science Foundation of China (Grant No. 81973814 and No. 81904132), the Natural Science Foundation of Guangdong Province (Grant No. 2017A030310129), the Natural Science Foundation of Guangdong Province (Grant No. 2020A1515010589), the Liu Xiaohong Famous Traditional Chinese Medicine Inheritance Studio from the Traditional Chinese Medicine Bureau of Guangdong Province (Grant No. 201805), the Construction Project of Respiratory Department National Clinical Medical Research Center (Grant No. 2110200309), 2018 Guangzhou University of Chinese Medicine National University Student Innovation and Entrepreneurship Training Project (Grant No. 201810572038), 2020 National College Student Innovation and Entrepreneurship Training Project of Guangzhou University of Chinese Medicine (Grant No. 202010572001), the Student Learning Team Incubation Project of Innovation Academy from The First Affiliated Hospital of Guangzhou University of Chinese Medicine (Grant No. 2018XXTD003), and the Technology Research of COVID-19 Treatment and Prevention and Special Project of Traditional Chinese Medicine Application-Research on the platform construction for the prevention and treatment of viral infectious diseases with traditional Chinese medicine (Grant No. 2020KJCX-KTYJ-130).

## Conflict of Interest

The authors declare that the research was conducted in the absence of any commercial or financial relationships that could be construed as a potential conflict of interest.

## Publisher’s Note

All claims expressed in this article are solely those of the authors and do not necessarily represent those of their affiliated organizations, or those of the publisher, the editors and the reviewers. Any product that may be evaluated in this article, or claim that may be made by its manufacturer, is not guaranteed or endorsed by the publisher.

## References

[1] Wiersinga WJ Rhodes A Cheng AC Peacock SJ Prescott HC. Pathophysiology, Transmission, Diagnosis, and Treatment of Coronavirus Disease 2019 (COVID-19): A Review. JAMA (2020) 324(8):782–93. 10.1001/jama.2020.12839 32648899

[2] Guzman MG Harris E. Dengue. Lancet (2015) 385(9966):453–65. 10.1016/S0140-6736(14)60572-9 25230594

[3] Harapan H Ryan M Yohan B Abidin RS Nainu F Rakib A. Covid-19 and Dengue: Double Punches for Dengue-Endemic Countries in Asia. Rev Med Virol (2021) 31(2):e2161. 10.1002/rmv.2161 32946149 PMC7536968

[4] Lustig Y Keler S Kolodny R Ben-Tal N Atias-Varon D Shlush E. Potential Antigenic Cross-Reactivity Between SARS-CoV-2 and Dengue Viruses. Clin Infect Dis (2020). 10.1093/cid/ciaa1207 PMC7454334 32797228

[5] Kembuan GJ. Dengue Serology in Indonesian COVID-19 Patients: Coinfection or Serological Overlap? IDCases (2020) 22:e00927. 10.1016/j.idcr.2020.e00927 32802747 PMC7403131

[6] Yan G Lee CK Lam LTM Yan B Chua YX Lim AYN. Covert COVID-19 and False-Positive Dengue Serology in Singapore, The Lancet. Infect Dis (2020) 20(5):536. 10.1016/S1473-3099(20)30158-4 PMC7128937 32145189

[7] Epelboin L Blondé R Nacher M Combe P Collet L. COVID-19 and Dengue Co-Infection in a Returning Traveller. J Travel Med (2020) 27(6). 10.1093/jtm/taaa114 PMC7454756 32657339

[8] Saavedra-Velasco M Chiara-Chilet C Pichardo-Rodriguez R Grandez-Urbina A Inga-Berrospi F. Coinfection Between Dengue and Covid-19: Need for Approach in Endemic Zones. Rev Fac Cien Med (Cordoba Argentina) (2020) 77(1):52–4. 10.31053/1853.0605.v77.n1.28031 32238260

[9] Carosella LM Pryluka D Maranzana A Barcan L Cuini R Freuler C. Characteristics of Patients Co-Infected With Severe Acute Respiratory Syndrome Coronavirus 2 and Dengue Virus, Buenos Aires, Argentina, March-June 2020. Emerg Infect Dis (2021) 27(2):348–51. 10.3201/eid2702.203439 PMC7853556 33347804

[10] Cardona-Ospina JA Arteaga-Livias K Villamil-Gomez WE Perez-Diaz CE Katterine Bonilla-Aldana D Mondragon-Cardona A. Dengue and COVID-19, Overlapping Epidemics? An Analysis From Colombia. J Med Virol (2021) 93(1):522–7. 10.1002/jmv.26194 PMC7323437 32558962

[11] Lorenz C Azevedo TS Chiaravalloti-Neto F. COVID-19 and Dengue Fever: A Dangerous Combination for the Health System in Brazil. Travel Med Infect Dis (2020) 35:101659. 10.1016/j.tmaid.2020.101659 32278756 PMC7144614

[12] Haqqi A Awan UA Ali M Saqib MAN Ahmed H Afzal MS. COVID-19 and Dengue Virus Coepidemics in Pakistan: A Dangerous Combination for an Overburdened Healthcare System. J Med Virol (2021) 93(1):80–2. 10.1002/jmv.26144 PMC7300443 32510175

[13] Nacher M Douine M Gaillet M Flamand C Rousset D Rousseau C. Simultaneous Dengue and COVID-19 Epidemics: Difficult Days Ahead? PloS Negl Trop Dis (2020) 14(8):e0008426. 10.1371/journal.pntd.0008426 32797035 PMC7428060

[14] Wilder-Smith A Tissera H Ooi EE Coloma J Scott TW Gubler DJ. Preventing Dengue Epidemics During the COVID-19 Pandemic. Am J Trop Med Hyg (2020) 103(2):570–1. 10.4269/ajtmh.20-0480 PMC7410414 32539912

[15] Blanco-Melo D Nilsson-Payant BE Liu WC Uhl S Hoagland D Møller R. Imbalanced Host Response to SARS-CoV-2 Drives Development of COVID-19. Cell (2020) 181(5):1036–45.e9. 10.1016/j.cell.2020.04.026 32416070 PMC7227586

[16] Grant RA Morales-Nebreda L Markov NS Swaminathan S Querrey M Guzman ER. Circuits Between Infected Macrophages and T Cells in SARS-CoV-2 Pneumonia. Nature (2021) 590(7847):635–41. 10.1038/s41586-020-03148-w PMC7987233 33429418

[17] Overmyer KA Shishkova E Miller IJ Balnis J Bernstein MN Peters-Clarke TM. Large-Scale Multi-Omic Analysis of COVID-19 Severity. Cell Syst (2021) 12(1):23–40.e7. 10.1016/j.cels.2020.10.003 33096026 PMC7543711

[18] Wang Y Suzek T Zhang J Wang J He S Cheng T. PubChem BioAssay: 2014 Update. Nucleic Acids Res (2014) 42(Database issue):D1075–82. 10.1093/nar/gkt978 PMC3965008 24198245

[19] Davis AP Grondin CJ Johnson RJ Sciaky D McMorran R Wiegers J. The Comparative Toxicogenomics Database: Update 2019. Nucleic Acids Res (2019) 47(D1):D948–54. 10.1093/nar/gky868 30247620 PMC6323936

[20] Piñero J Bravo À Queralt-Rosinach N Gutiérrez-Sacristán A Deu-Pons J Centeno E. DisGeNET: A Comprehensive Platform Integrating Information on Human Disease-Associated Genes and Variants. Nucleic Acids Res (2017) 45(D1):D833–39. 10.1093/nar/gkw943 27924018 PMC5210640

[21] Popper SJ Gordon A Liu M Balmaseda A Harris E Relman DA. Temporal Dynamics of the Transcriptional Response to Dengue Virus Infection in Nicaraguan Children. PloS Negl Trop Dis (2012) 6(12):e1966. 10.1371/journal.pntd.0001966 23285306 PMC3527342

[22] Kwissa M Nakaya HI Onlamoon N Wrammert J Villinger F Perng GC. Dengue Virus Infection Induces Expansion of a CD14(+)CD16(+) Monocyte Population That Stimulates Plasmablast Differentiation. Cell Host Microbe (2014) 16(1):115–27. 10.1016/j.chom.2014.06.001 PMC4116428 24981333

[23] Chandele A Sewatanon J Gunisetty S Singla M Onlamoon N Akondy RS. Characterization of Human CD8 T Cell Responses in Dengue Virus-Infected Patients From India. J Virol (2016) 90(24):11259–78. 10.1128/JVI.01424-16 PMC5126381 27707928

[24] Safran M Dalah I Alexander J Rosen N Iny Stein T Shmoish M. GeneCards Version 3: The Human Gene Integrator. Database (2010) 2010:baq020. 10.1093/database/baq020 20689021 PMC2938269

[25] Szklarczyk D Gable AL Lyon D Junge A Wyder S Huerta-Cepas J. STRING V11: Protein-Protein Association Networks With Increased Coverage, Supporting Functional Discovery in Genome-Wide Experimental Datasets. Nucleic Acids Res (2019) 47(D1):D607–13. 10.1093/nar/gky1131 30476243 PMC6323986

[26] Shannon P Markiel A Ozier O Baliga NS Wang JT Ramage D. Cytoscape: A Software Environment for Integrated Models of Biomolecular Interaction Networks. Genome Res (2003) 13(11):2498–504. 10.1101/gr.1239303 PMC403769 14597658

[27] Bader GD Hogue CW. An Automated Method for Finding Molecular Complexes in Large Protein Interaction Networks. BMC Bioinformatics (2003) 4:2. 10.1186/1471-2105-4-2 12525261 PMC149346

[28] Chin CH Chen SH Wu HH Ho CW Ko MT Lin CY. cytoHubba: Identifying Hub Objects and Sub-Networks From Complex Interactome. BMC Syst Biol (2014) 8 Suppl 4(Suppl 4):S11. 10.1186/1752-0509-8-S4-S11 25521941 PMC4290687

[29] Zhou Y Zhou B Pache L Chang M Khodabakhshi AH Tanaseichuk O. Metascape Provides a Biologist-Oriented Resource for the Analysis of Systems-Level Datasets. Nat Commun (2019) 10(1):1523. 10.1038/s41467-019-09234-6 30944313 PMC6447622

[30] Rydenfelt M Klinger B Klunemann M Bluthgen N. SPEED2: Inferring Upstream Pathway Activity From Differential Gene Expression. Nucleic Acids Res (2020) 48(W1):W307–12. 10.1093/nar/gkaa236 PMC7319438 32313938

[31] GTEx Consortium. The Genotype-Tissue Expression (GTEx) Project. Nat Genet (2013) 45(6):580–5. 10.1038/ng.2653 PMC4010069 23715323

[32] Human genomics. The Genotype-Tissue Expression (GTEx) Pilot Analysis: Multitissue Gene Regulation in Humans. Science (New York N Y) (2015) 348(6235):648–60. 10.1126/science.1262110 PMC4547484 25954001

[33] Xiong Y Liu Y Cao L Wang D Guo M Jiang A. Transcriptomic Characteristics of Bronchoalveolar Lavage Fluid and Peripheral Blood Mononuclear Cells in COVID-19 Patients. Emerg Microbes Infect (2020) 9(1):761–70. 10.1080/22221751.2020.1747363 PMC7170362 32228226

[34] Chevrier S Zurbuchen Y Cervia C Adamo S Raeber ME de Souza N. A Distinct Innate Immune Signature Marks Progression From Mild to Severe COVID-19. Cell Rep Med (2021) 2(1):100166. 10.1016/j.xcrm.2020.100166 33521697 PMC7817872

[35] Sprokholt JK Kaptein TM van Hamme JL Overmars RJ Gringhuis SI Geijtenbeek TBH. RIG-I-Like Receptor Triggering by Dengue Virus Drives Dendritic Cell Immune Activation and TH1 Differentiation. J Immunol (2017) 198(12):4764–71. 10.4049/jimmunol.1602121 28507028

[36] Matsuyama T Kubli SP Yoshinaga SK Pfeffer K Mak TW. An Aberrant STAT Pathway Is Central to COVID-19. Cell Death Differ (2020) 27(12):3209–25. 10.1038/s41418-020-00633-7 PMC7545020 33037393

[37] Chao CH Wu WC Lai YC Tsai PJ Perng GC Lin YS. Dengue Virus Nonstructural Protein 1 Activates Platelets *Via* Toll-Like Receptor 4, Leading to Thrombocytopenia and Hemorrhage. PloS Pathog (2019) 15(4):e1007625. 10.1371/journal.ppat.1007625 31009511 PMC6497319

[38] Liu Y Lv J Liu J Li M Xie J Lv Q. Mucus Production Stimulated by IFN-AhR Signaling Triggers Hypoxia of COVID-19. Cell Res (2020) 30(12):1078–87. 10.1038/s41422-020-00435-z PMC7646495 33159154

[39] Giovannoni F Bosch I Polonio CM Torti MF Wheeler MA Li Z. AHR Is a Zika Virus Host Factor and a Candidate Target for Antiviral Therapy. Nat Neurosci (2020) 23(8):939–51. 10.1038/s41593-020-0664-0 PMC7897397 32690969

[40] Priyadarshini D Gadia RR Tripathy A Gurukumar KR Bhagat A Patwardhan S. Clinical Findings and Pro-Inflammatory Cytokines in Dengue Patients in Western India: A Facility-Based Study. PloS One (2010) 5(1):e8709. 10.1371/journal.pone.0008709 20090849 PMC2806829

[41] Moore JB June CH. Cytokine Release Syndrome in Severe COVID-19. Science (2020) 368(6490):473–4. 10.1126/science.abb8925 32303591

[42] Li X Ma X. Acute Respiratory Failure in COVID-19: Is it “Typical” ARDS? Crit Care (2020) 24(1):198. 10.1186/s13054-020-02911-9 32375845 PMC7202792

[43] Juffrie M Meer GM Hack CE Haasnoot K Sutaryo AJ Veerman LG. Thijs, Inflammatory Mediators in Dengue Virus Infection in Children: Interleukin-6 and its Relation to C-Reactive Protein and Secretory Phospholipase A2. Am J Trop Med Hyg (2001) 65(1):70–5. 10.4269/ajtmh.2001.65.70 11504411

[44] Dayarathna S Jeewandara C Gomes L Somathilaka G Jayathilaka D Vimalachandran V. Similarities and Differences Between the ‘Cytokine Storms’ in Acute Dengue and COVID-19. Sci Rep (2020) 10(1):19839. 10.1038/s41598-020-76836-2 33199778 PMC7670444

[45] Fung TS Liu DX. Activation of the C-Jun NH2-Terminal Kinase Pathway by Coronavirus Infectious Bronchitis Virus Promotes Apoptosis Independently of C-Jun. Cell Death Dis (2017) 8(12):3215. 10.1038/s41419-017-0053-0 29238080 PMC5870581

[46] Ye Z Wong CK Li P Xie Y. A SARS-CoV Protein, ORF-6, Induces Caspase-3 Mediated, ER Stress and JNK-Dependent Apoptosis. Biochim Biophys Acta (2008) 1780(12):1383–7. 10.1016/j.bbagen.2008.07.009 PMC7115782 18708124

[47] Fung TS Liu DX. Coronavirus Infection, ER Stress, Apoptosis and Innate Immunity. Front Microbiol (2014) 5:296. 10.3389/fmicb.2014.00296 24987391 PMC4060729

[48] Sim S Jupatanakul N Ramirez JL Kang S Romero-Vivas CM Mohammed H. Transcriptomic Profiling of Diverse Aedes Aegypti Strains Reveals Increased Basal-Level Immune Activation in Dengue Virus-Refractory Populations and Identifies Novel Virus-Vector Molecular Interactions. PloS Negl Trop Dis (2013) 7(7):e2295. 10.1371/journal.pntd.0002295 23861987 PMC3701703

[49] Chowdhury A Modahl CM Tan ST Wong Wei Xiang B Misse D Vial T. JNK Pathway Restricts DENV2, ZIKV and CHIKV Infection by Activating Complement and Apoptosis in Mosquito Salivary Glands. PloS Pathog (2020) 16(8):e1008754. 10.1371/journal.ppat.1008754 32776975 PMC7444518

[50] Yao Z Zheng Z Wu K Junhua Z. Immune Environment Modulation in Pneumonia Patients Caused by Coronavirus: SARS-CoV, MERS-CoV and SARS-CoV-2. Aging (Albany NY) (2020) 12(9):7639–51. 10.18632/aging.103101 PMC7244084 32364527

[51] Platanias LC. Mechanisms of Type-I- and Type-II-Interferon-Mediated Signalling. Nat Rev Immunol (2005) 5(5):375–86. 10.1038/nri1604 15864272

[52] Catanzaro M Fagiani F Racchi M Corsini E Govoni S Lanni C. Immune Response in COVID-19: Addressing a Pharmacological Challenge by Targeting Pathways Triggered by SARS-CoV-2. Signal Transduct Target Ther (2020) 5(1):84. 10.1038/s41392-020-0191-1 32467561 PMC7255975

[53] Nasirudeen AM Wong HH Thien P Xu S Lam KP Liu DX. RIG-I, MDA5 and TLR3 Synergistically Play an Important Role in Restriction of Dengue Virus Infection. PloS Negl Trop Dis (2011) 5(1):e926. 10.1371/journal.pntd.0000926 21245912 PMC3014945

[54] Tsai YT Chang SY Lee CN Kao CL. Human TLR3 Recognizes Dengue Virus and Modulates Viral Replication *In Vitro* . Cell Microbiol (2009) 11(4):604–15. 10.1111/j.1462-5822.2008.01277.x 19134117

[55] Korakas E Ikonomidis I Kousathana F Balampanis K Kountouri A Raptis A. Obesity and COVID-19: Immune and Metabolic Derangement as a Possible Link to Adverse Clinical Outcomes. Am J Physiol Endocrinol Metab (2020) 319(1):E105–9. 10.1152/ajpendo.00198.2020 PMC7322508 32459524

[56] Freeman TL Swartz TH. Targeting the NLRP3 Inflammasome in Severe COVID-19. Front Immunol (2020) 11:1518. 10.3389/fimmu.2020.01518 32655582 PMC7324760

[57] Shrivastava G Visoso-Carvajal G Garcia-Cordero J Leon-Juarez M Chavez-Munguia B Lopez T. Dengue Virus Serotype 2 and Its Non-Structural Proteins 2A and 2B Activate NLRP3 Inflammasome. Front Immunol (2020) 11:352. 10.3389/fimmu.2020.00352 32210961 PMC7076137

[58] Pan P Zhang Q Liu W Wang W Lao Z Zhang W. Dengue Virus M Protein Promotes NLRP3 Inflammasome Activation To Induce Vascular Leakage in Mice. J Virol (2019) 93(21):1–19. 10.1128/JVI.00996-19 PMC6803285 31413130

[59] Gabay C Lamacchia C Palmer G. IL-1 Pathways in Inflammation and Human Diseases. Nat Rev Rheumatol (2010) 6(4):232–41. 10.1038/nrrheum.2010.4 20177398

[60] Shaath H Vishnubalaji R Elkord E Alajez NM. Single-Cell Transcriptome Analysis Highlights a Role for Neutrophils and Inflammatory Macrophages in the Pathogenesis of Severe COVID-19. Cells (2020) 9(11):1–19. 10.3390/cells9112374 PMC7693119 33138195

[61] Hottz ED Lopes JF Freitas C Valls-de-Souza R Oliveira MF Bozza MT. Platelets Mediate Increased Endothelium Permeability in Dengue Through NLRP3-Inflammasome Activation. Blood (2013) 122(20):3405–14. 10.1182/blood-2013-05-504449 PMC3829114 24009231

[62] Sa-Ngasang A Ohashi J Naka I Anantapreecha S Sawanpanyalert P Patarapotikul J. Association of IL1B -31C/T and IL1RA Variable Number of an 86-Bp Tandem Repeat With Dengue Shock Syndrome in Thailand. J Infect Dis (2014) 210(1):138–45. 10.1093/infdis/jiu042 24446526

[63] Hadjadj J Yatim N Barnabei L Corneau A Boussier J Smith N. Impaired Type I Interferon Activity and Inflammatory Responses in Severe COVID-19 Patients. Science (2020) 369(6504):718–24. 10.1126/science.abc6027 PMC7402632 32661059

[64] Karki R Sharma BR Tuladhar S Williams EP Zalduondo L Samir P. Synergism of TNF-Alpha and IFN-Gamma Triggers Inflammatory Cell Death, Tissue Damage, and Mortality in SARS-CoV-2 Infection and Cytokine Shock Syndromes. Cell (2021) 184(1):149–68.e17. 10.1016/j.cell.2020.11.025 33278357 PMC7674074

[65] Bethell DB Flobbe K Cao XT Day NP Pham TP Buurman WA. Pathophysiologic and Prognostic Role of Cytokines in Dengue Hemorrhagic Fever. J Infect Dis (1998) 177(3):778–82. 10.1086/517807 9498463

[66] Yen YT Chen HC Lin YD Shieh CC Wu-Hsieh BA. Enhancement by Tumor Necrosis Factor Alpha of Dengue Virus-Induced Endothelial Cell Production of Reactive Nitrogen and Oxygen Species Is Key to Hemorrhage Development. J Virol (2008) 82(24):12312–24. 10.1128/JVI.00968-08 PMC2593312 18842737

[67] Chen HC Hofman FM Kung JT Lin YD Wu-Hsieh BA. Both Virus and Tumor Necrosis Factor Alpha Are Critical for Endothelium Damage in a Mouse Model of Dengue Virus-Induced Hemorrhage. J Virol (2007) 81(11):5518–26. 10.1128/JVI.02575-06 PMC1900309 17360740

[68] Tong M Jiang Y Xia D Xiong Y Zheng Q Chen F. Elevated Expression of Serum Endothelial Cell Adhesion Molecules in COVID-19 Patients. J Infect Dis (2020) 222(6):894–8. 10.1093/infdis/jiaa349 PMC7337874 32582936

[69] Vitoria WO Thome LS Kanashiro-Galo L Carvalho LV Penny R Santos WLC. Upregulation of Intercellular Adhesion Molecule-1 and Vascular Cell Adhesion Molecule-1 in Renal Tissue in Severe Dengue in Humans: Effects on Endothelial Activation/Dysfunction. Rev Soc Bras Med Trop (2019) 52:e20180353. 10.1590/0037-8682-0353-2018 31778418

[70] Zheng W Wu H Wang T Zhan S Liu X. Quercetin for COVID-19 and DENGUE Co-Infection: A Potential Therapeutic Strategy of Targeting Critical Host Signal Pathways Triggered by SARS-CoV-2 and DENV. Brief Bioinform (2021) 8:1–33. 10.1093/bib/bbab199 PMC8195157 34058750

[71] Zainal N Chang CP Cheng YL Wu YW Anderson R Wan SW. Resveratrol Treatment Reveals a Novel Role for HMGB1 in Regulation of the Type 1 Interferon Response in Dengue Virus Infection. Sci Rep (2017) 7:42998. 10.1038/srep42998 28216632 PMC5316936

[72] Ferreira AO Polonini HC Dijkers ECF. Postulated Adjuvant Therapeutic Strategies for COVID-19. J Pers Med (2020) 10(3):1–33. 10.3390/jpm10030080 PMC7565841 32764275

[73] Malaguarnera L. Influence of Resveratrol on the Immune Response. Nutrients (2019) 11(5):1–24. 10.3390/nu11050946 PMC6566902 31035454

[74] Ungvari Z Bagi Z Feher A Recchia FA Sonntag WE Pearson K. Resveratrol Confers Endothelial Protection *Via* Activation of the Antioxidant Transcription Factor Nrf2. Am J Physiol Heart Circ Physiol (2010) 299(1):H18–24. 10.1152/ajpheart.00260.2010 PMC2904129 20418481

[75] Lou Z Du K Wang T Zhao X Li X Wang B. Resveratrol Suppresses P-Selectin, PSGL-1, and VWF Through SIRT1 Signaling Pathway. Acta Biochim Biophys Sin (Shanghai) (2017) 49(9):848–50. 10.1093/abbs/gmx077 28910974

[76] Vogt I Mestres J. Information Loss in Network Pharmacology. Mol Inform (2019) 38(7):e1900032. 10.1002/minf.201900032 30957433

[77] Corwin J Nonneman A Goodlett C. Limited Sparing a Function on Spatial Delayed Alternation After Two-Stage Lesions of Prefrontal Cortex in the Rat. Physiol Behav (1981) 26(5):763–71. 10.1016/0031-9384(81)90096-2 7267769

